# Assessment of the speed of flea kill of lotilaner (Credelio™) throughout the month following oral administration to dogs

**DOI:** 10.1186/s13071-017-2466-0

**Published:** 2017-11-01

**Authors:** Daniela Cavalleri, Martin Murphy, Wolfgang Seewald, Jason Drake, Steve Nanchen

**Affiliations:** 1 Elanco Animal Health, Mattenstrasse 24a, 4058 Basel, Switzerland; 2 Elanco Animal Health, 2500 Innovation Way, Greenfield, IN 46140 USA

**Keywords:** Fleas, *Ctenocephalides felis*, Lotilaner, Credelio™, Speed of kill, Dog, Oral

## Abstract

**Background:**

Lotilaner (Credelio™, Elanco), a novel isoxazoline, is a systemic insecticide and acaricide that is rapidly absorbed following oral administration to dogs and has a half-life of 30 days. As part of a development program, studies were undertaken to investigate lotilaner’s initial and sustained efficacy and speed of kill against fleas.

**Methods:**

Four studies were conducted to evaluate the onset of lotilaner’s speed of flea knockdown at the time of treatment, and to determine the sustained speed of flea kill (SOK) up to 35 days post-treatment. Each study assessed one or two specific time points (4, 6, 8 and 12 h) post-treatment and following weekly re-infestations. In each study, dogs were randomised to a lotilaner or an untreated group based on pre-administration flea counts, and before treatment were infested with adult *Ctenocephalides felis*. Dogs randomised to a lotilaner group received a single treatment on Day 0, at the minimum recommended dose rate of 20 mg/kg, 30 (± 5) minutes after being fed. Efficacy was calculated using geometric, and arithmetic mean flea counts in treated versus untreated groups.

**Results:**

On Day 0, lotilaner efficacy was 89.9% at 4 h, 99.2% at 6 h, 99.9% at 8 h, and 100% at 12 h post-treatment. At each weekly assessment, lotilaner efficacy at 4 h remained at > 97%, at 8 h remained at > 99%, and at 12 h remained at 100% through Day 35. Across all studies, there were no treatment-related adverse events.

**Conclusion:**

Lotilaner’s rapid flea knockdown immediately following treatment and sustained SOK through 35 days post-treatment offers a new solution for helping to eliminate the health risks that accompany flea infestations on dogs. The consistency of the rapid, sustained flea SOK demonstrated in these studies generates confidence that monthly use of lotilaner in dogs can be valuable in disrupting the flea life cycle in a contaminated environment, and that newly acquired fleas will die quickly, thereby reducing the discomfort of flea bites. The sustained lotilaner SOK also provides confidence that there will be no “end-of-dose” resurgence in flea burdens with the potential accompanying consequence of flares in flea-bite hypersensitivity.

**Electronic supplementary material:**

The online version of this article (10.1186/s13071-017-2466-0) contains supplementary material, which is available to authorized users.

## Background

Approaches to monthly canine flea control have undergone major changes during the last 10 years, accelerating a trend away from the application of spot-on ectoparasiticides in favour of a range of orally administered products. Early work demonstrated that orally administered products have the potential to kill fleas more quickly than those that act topically, perhaps because of the time required for a topical product to spread from the application site to distal parts of the animal [1, 2]. The emergence of orally administered flea-killing products has also brought attention to other potential shortcomings of topical products. These include concern about household pesticide exposure, the requirement for careful application by owners directly to a pet’s skin, and the decline in skin concentrations over the month following treatment, which may vary from animal to animal, and be unpredictable under different climatic or water exposure conditions [3].

The first monthly oral flea adulticide product for dogs, spinosad, has been shown to provide a rapid onset of flea knockdown while providing a month of flea-killing activity [4]. The rapid killing of fleas was seen as desirable, not only to break the flea life cycle by preventing flea egg production, but also to quickly remove a source of irritation from biting fleas, a cause of allergic dermatitis, and to quickly kill fleas that are known to transmit a number of infectious agents such as *Dipylidium caninum, Acanthocheilonema reconditum*, *Bartonella* spp. and *Rickettsia* spp. [5].

A more recent innovation in canine ectoparasite control has been the emergence of a novel family of compounds, the isoxazolines, that when administered orally have been shown to provide sustained activity against fleas, as well as against common species of ticks [6–8]. The first of these compounds to be approved, in 2014, were afoxolaner and fluralaner, followed in 2016 by sarolaner. The isoxazolines have been shown to have a novel mode of action, binding at chloride ion channels that are gated by γ-aminobutyric acid (GABA) and glutamate receptors, leading to a progressive and irreversible paralysis in insects and acarines [8, 9]. The binding site of the isoxazolines is different from that of other insecticides and acaricides, and cross-resistance is, therefore, unlikely to develop [8, 9].

The most recent isoxazoline to be approved for use in dogs is lotilaner. The lotilaner molecule was selected from a library of more than 500 compounds because of its potential to provide a rapid and sustained knockdown of insects and acarines. Following its selection and preliminary studies, lotilaner was formulated for development as a flavoured chewable tablet (Credelio™, Elanco). Preclinical investigations included a study in which lotilaner was shown to be safe when administered on eight consecutive monthly occasions, at the highest point of the label dose range (20 to 43 mg/kg), and at three- and five- times that dose to puppies from 8 weeks of age [10]. A pharmacokinetic study found that when administered with food, lotilaner is rapidly absorbed, with peak plasma values occurring within approximately 2 h following oral administration, and has a half-life of approximately 30 days [11].

These characteristics fueled expectations that lotilaner would provide a rapid knockdown of fleas and ticks, and that that rapid knockdown could be sustained for at least 1 month following administration, avoiding any concern of an end-of-dose tapering of effectiveness. To assess the alignment of product attribute expectations with reality, a series of four studies was undertaken to investigate the efficacy of lotilaner against fleas (*Ctenocephalides felis*). These studies were designed to provide insight into lotilaner’s speed of kill (SOK) of fleas present at the time of treatment and up to 35 days following treatment.

While the onset of action of flea control products is important to quickly alleviate the irritation caused by existing infestations, it is also important for that speed of kill (SOK) to be sustained through the full labelled dose period. The potential for an end-of-dose reduction in the speed of flea kill has been reported for some products as concentrations of the active ingredient decline to levels below those needed to ensure rapid insecticidal effectiveness [12–14].

## Methods

The studies reported in this paper were undertaken at four laboratories in three countries. All personnel conducting study observations and counts or performing infestations were blinded to treatment allocations.

### Animals

Dogs were healthy pure-bred male and female Beagles, aged between 7 months and 9 years and ranging in weights from 6.8 to 19.6 kg. The dogs that were enrolled were selected from a larger group that had been acclimated to the respective study facility, and as needed all had undergone a washout period sufficient to ensure that there was no residual parasiticide present that could affect study results. In all studies, dogs were housed in individual pens, with no direct contact possible between individual dogs. Standard precautions were undertaken to ensure that there was no cross-contamination between dogs within or between treatment groups. Dogs were provided food and water according to each facility’s standard procedure, and health observations were made at least once daily throughout pre-study and study periods. At the end of the study, dogs were returned to the study kennels.

Throughout each study, observations were made to determine and record the occurrence of any adverse event. An adverse event was defined as any observation of any clinical sign in any dog that was unfavourable and unintended and occurred after the use of a product, whether or not such an event was considered to be product related [15].

### Design

All studies utilised a randomised, blinded, negative-controlled design. For randomization purposes, dogs were infested with up to approximately 100 fleas between 6 days and 2 weeks before the start of each study, and fleas were combed out and counted 24 or 48 h post-infestation. These counts were used to rank dogs, with those not retaining at least 50% of the infestation being ineligible for study inclusion. As there is no effect of sex on lotilaner systemic absorption, this was not a factor in ranking and randomization [10]. Ranking was used to place dogs into blocks, and within each block, dogs were randomised to a treated or a control group. In all studies, the comparator was a sham-treated or untreated control group, and all dogs consumed a partial daily ration within approximately 30 min before treatment was administered. There were eight dogs included in each study group within the four studies.

### Treatment, flea infestations and combing

Each study included at least one group in which lotilaner was administered orally at a minimum dose of 20 mg/kg and at least one corresponding control group that received no active antiparasitic product. All dogs had consumed a partial daily ration within approximately 30 min before dosing. The number of groups in each study was fixed according to the number of time intervals at which assessments were to be undertaken. Contemporaneous control groups were used at each time point assessed.

- *Study 1*: One lotilaner-treated group, and one control group. Flea counts were undertaken 4 h post-dosing against an existing infestation and 4 h after new infestations on days 7, 14, 21, 28 and 35.
- *Study 2*: One lotilaner-treated group, and one control group. Flea counts at 6 h post-dosing against an existing infestation and 6 h after new infestations on days 7, 14, 21, 28 and 35.
- *Study 3*: Two lotilaner-treated groups, and two control groups. Flea counts at eight and 12 h post-dosing against an existing flea infestation and eight and 12 h after new infestations on days 7, 14, 21, 28 and 35.
- *Study 4*: Two lotilaner-treated groups, and two control groups. Flea counts at 12 and 24 h post-dosing against an existing flea infestation and 12 and 24 h after new infestations on days 7, 14, 21, 28 and 35.

Flea infestations were performed according to each laboratory’s standard procedure, typically involving application from a vial containing 100 recently hatched, unfed, adult fleas to the base of a dog’s tail or a dog’s flank. Flea strains originated from Ireland, Germany-Denmark and the United States. At protocol-specified times, thorough flea combing using fine-toothed flea combs was completed to determine product efficacy. Techniques of flea retrieval were applied according to each laboratory’s standard procedure, with some variation between laboratories. In two studies, combing was required for at least 5 min, extending beyond that time if fleas continued to be found. In two studies combing was required minimally for 10 min, and extended by 2 min as additional fleas were found, to a maximum of 16 min.

In all studies, recovered fleas were classified as live or dead; a flea was classified as live if it could actively move through hair, and if placed on a flat surface, it rapidly “righted” itself and readily moved or jumped. In studies 1 through 3, any fleas that were laterally recumbent, could not normally move through hair or “right” themselves when placed on a flat surface, but still had leg movement or twitching, were recorded as moribund but included in the live flea counts. A flea was classified as dead if it was completely immobile. In Study 4 moribund fleas were not counted separately and were included as dead.

### Assessments of efficacy

The individual dog was the experimental unit. Using the formula below, efficacy against fleas was calculated separately for each count time point. All analyses were carried out using the PROC MIXED procedure (SAS 9.2, Cary NC). Geometric means were calculated using logarithm transformed counts (count +1) with one (1) subsequently subtracted from the result, and an ANOVA model was used to compare the treated and untreated groups.

$$ \mathrm{Efficacy}\ \left(\%\right)=100\times \left(\mathrm{Mc}\hbox{--} \mathrm{Mt}\right)/\mathrm{Mc} $$

where Mc is the mean number of live fleas in the untreated control group at each time point on each day, and Mt is the mean number of live fleas in the corresponding treated group at each time point on each day.

Treatment was considered effective at any assessment if at least six animals in respective control groups had a recovery rate of at least approximately 50%, if relative to the control group the percent reduction in mean flea counts in the treated group was at least 90% (or 95%, depending on regulatory requirement), and if the reduction was statistically significant (*P* < 0.05).

### Translations

Spanish translation of the article is available in Additional file 1. French translation of the Abstract is available in Additional file 2.

## Results

**Fig. 1:**
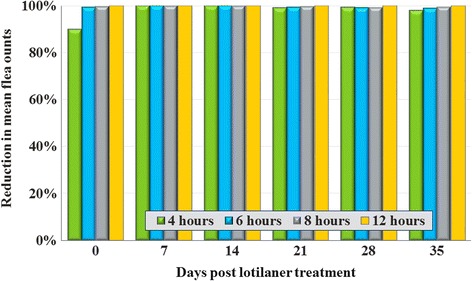
Percent reductions in geometric mean flea counts for lotilaner compared to untreated controls at 4, 6, 8 and 12 h post-treatment and at the same intervals following weekly infestations through 35 days after treatment

Infestations in the control groups at each time point and in each study were adequate. At 4 h post-treatment, efficacy based on geometric means was 89.9%, and 4 h following subsequent infestations ranged from 97.8 to 100% up to and through Day 35. At 8 h post-treatment and after new infestations, lotilaner efficacy was at least 99.5% up to and through Day 35, and at 12 h was 100% at all assessments through Day 35 (Fig. 1; Tables 1, 2, 3, 4, 5 and 6).

**Table 1:** Results of flea infestations at 4 h post-treatment and 4 h after weekly infestations in Study 1

		Day of infestation and flea count					
		0	7	14	21	28	35
Control	Arithmetic mean ± SD	60.9 ± 12.5	76.0 ± 9.8	78.6 ± 13.1	71.0 ± 6.4	67.3 ± 5.6	73.1 ± 12.3
	Geometric mean	59.9	75.4	77.7	70.8	67.1	72.3
Lotilaner	Arithmetic mean ± SD	26.4 ± 33.8	0.1 ± 0.4	0.0 ± 0.0	1.0 ± 1.3	0.8 ± 1.2	2.1 ± 1.7
	Efficacy (%)	56.7	99.8	100.0	98.6	98.9	97.1
	Geometric mean	6.1	0.1	0.0	0.7	0.5	1.6
	Efficacy (%)	89.9	99.9	100	99.0	99.3	97.8
Comparison		*t*_(5)_ = 3.4, *P* = 0.0194	*t*_(5)_ = 55.0, *P* < 0.0001	*t*_(5)_ = 91.5, *P* < 0.0001	*t*_(5)_ = 21.8, *P* < 0.0001	*t*_(5)_ = 18.5, *P* < 0.0001	*t*_(5)_ = 14.9, *P* < 0.0001

*Abbreviation*: *SD* standard deviation

**Table 2:** Results of flea infestations at 6 h post-treatment and 6 h after weekly infestations in Study 2

		Day of infestation and flea count					
		0	7	14	21	28	35
Control	Arithmetic mean ± SD	76.6 ± 20.1	78.6 ± 8.7	88.9 ± 12.2	95.4 ± 5.6	94.5 ± 7.6	93.8 ± 7.9
	Geometric mean	74.3	78.2	88.1	95.2	94.2	93.5
Lotilaner	Arithmetic mean ± SD	2.6 ± 7.0	0.1 ± 0.4	0.0 ± 0.0	3.6 ± 10.3	1.6 ± 3.1	1.8 ± 2.4
	Efficacy (%)	96.6	99.8	100	96.2	98.3	98.1
	Geometric mean	0.6	0.1	0.0	0.5	0.8	1.0
	Efficacy (%)	99.2	99.9	100	99.4	99.1	98.9
Comparison		*t*_(7)_ = 11.4, *P* < 0.0001	*t*_(7)_ = 45.0, *P* < 0.0001	*t*_(7)_ = 88.4, *P* < 0.0001	*t*_(7)_ = 9.7, *P* < 0.0001	*t*_(7)_ = 14.3, *P* < 0.0001	*t*_(7)_ = 13.6, *P* < 0.0001

*Abbreviation*: *SD* standard deviation

**Table 3:** Results of flea infestations at 8 h post-treatment and 8 h after weekly infestations in Study 3

		Day of infestation and flea count					
		0	7	14	21	28	35
Control	Arithmetic mean ± SD	75.1 ± 6.7	82.3 ± 7.8	80.8 ± 11.4	81.4 ± 13.8	78.9 ± 13.4	76.3 ± 7.4
	Geometric mean	74.9	81.9	80.0	80.4	77.9	75.9
Lotilaner	Arithmetic mean ± SD	0.1 ± 0.4	0.0 ± 0.0	0.0 ± 0.0	0.1 ± 0.4	0.5 ± 0.8	0.4 ± 0.7
	Efficacy (%)	99.8	100	100	99.8	99.4	99.5
	Geometric mean	0.1	0.0	0.0	0.1	0.4	0.3
	Efficacy (%)	99.9	100	100	99.9	99.5	99.7
Comparison		*t*_(5)_ = 46.0, *P* < 0.0001	*t*_(5)_ = 128.2, *P* < 0.0001	*t*_(5)_ = 40.4, *P* < 0.0001	*t*_(5)_ = 42.1, *P* < 0.0001	*t*_(5)_ = 12.3, *P* < 0.0001	*t*_(5)_ = 27.6, *P* < 0.0001

*Abbreviation*: *SD* standard deviation

**Table 4:** Results of flea infestations at 12 h post-treatment and 12 h after weekly infestations in Study 3

		Day of infestation and flea count					
		0	7	14	21	28	35
Control	Arithmetic mean ± SD	75.0 ± 7.0	71.9 ± 9.2	76.9 ± 12.8	78.0 ± 9.4	75.1 ± 6.2	82.0 ± 10.3
	Geometric mean	74.7	71.4	75.9	77.6	74.9	81.4
Lotilaner	Arithmetic mean ± SD	0.0 ± 0.0	0.0 ± 0.0	0.0 ± 0.0	0.0 ± 0.0	0.0 ± 0.0	0.0 ± 0.0
	Efficacy (%)	100	100	100	100	100	100
	Geometric mean	0.0	0.0	0.0	0.0	0.0	0.0
	Efficacy (%)	100	100	100	100	100	100
Comparison		*t*_(5)_ = 78.5, *P* < 0.0001	*t*_(5)_ = 92.6, *P* < 0.0001	*t*_(5)_ = 42.2, *P* < 0.0001	*t*_(5)_ = 112.6, *P* < 0.0001	*t*_(5)_ = 146.6, *P* < 0.0001	*t*_(5)_ = 99.0, *P* < 0.0001

*Abbreviation*: *SD* standard deviation

**Table 5:** Results of flea infestations at 12 h post-treatment and 12 h after weekly infestations in Study 4

		Day of infestation and flea count					
		0	7	14	21	28	35
Control	Arithmetic mean ± SD	89.5 ± 36.9	69.8 ± 18.0	75.1 ± 22.6	75.8 ± 22.6	76.5 ± 19.4	59.8 ± 10.1
	Geometric mean	83.6	67.9	72.6	73.1	74.1	59.1
Lotilaner	Arithmetic mean ± SD	0.0 ± 0.0	0.0 ± 0.0	0.0 ± 0.0	0.0 ± 0.0	0.0 ± 0.0	0.0 ± 0.0
	Efficacy (%)	100	100	100	100	100	100
	Geometric mean	0.0	0.0	0.0	0.0	0.0	0.0
	Efficacy (%)	100	100	100	100	100	100
Comparison		*t*_(7)_ = 32.1, *P* < 0.0001	*t*_(7)_ = 49.7, *P* < 0.0001	*t*_(7)_ = 44.7, *P* < 0.0001	*t*_(7)_ = 43.5, *P* < 0.0001	*t*_(7)_ = 43.9, *P* < 0.0001	*t*_(7)_ = 75.0, *P* < 0.0001

*Abbreviation*: *SD* standard deviation

**Table 6:** Results of flea infestations at 24 h post-treatment and 24 h after weekly infestations in Study 4

		Day of infestation (flea counts completed the next day)					
		0	7	14	21	28	35
Control	Arithmetic mean ± SD	87.6 ± 7.4	72.1 ± 15.6	76.1 ± 22.4	65.6 ± 14.9	66.9 ± 18.3	59.5 ± 10.3
	Geometric mean	87.4	70.7	73.6	64.3	65.2	58.6
Lotilaner	Arithmetic mean ± SD	0.0 ± 0.0	0.0 ± 0.0	0.1 ± 0.4	0.1 ± 0.3	0.0 ± 0.0	0.0 ± 0.0
	Efficacy (%)	100	100	99.8	99.8	100	100
	Geometric mean	0.0	0.0	0.1	0.1	0.0	0.0
	Efficacy (%)	100	100	99.8	99.8	100	100
Comparison		*t*_(7)_ = 152.3, *P* < 0.0001	*t*_(7)_ = 55.8, *P* < 0.0001	*t*_(7)_ = 38.7, *P* < 0.0001	*t*_(7)_ = 35.7, *P* < 0.0001	*t*_(7)_ = 52.4, *P* < 0.0001	*t*_(7)_ = 61.3, *P* < 0.0001

*Abbreviation*: *SD* standard deviation

### Study 1

Within 4 h after treatment, geometric mean flea counts had been reduced by 89.9% in lotilaner-treated dogs; and following all subsequent infestations, four-hour flea count reductions in both geometric and arithmetic means were within the efficacy range of 97.1 to 100% (Table 1). Moribund fleas were present on lotilaner-group dogs on all days, except on Day 14 when there were no fleas classified as either live or moribund. The numbers of moribund fleas on lotilaner-treated dogs varied across challenges, with a total of 20 on Day 0, and ranging from 1 to 14 on other days. Compared to the control group, mean flea count reductions for the lotilaner group were significant at every assessment through Day 35 (*t*_(5)_ = 3.4, *P* = 0.0194 on Day 0 and *t*_(5)_ ≥ 14.9, *P* < 0.0001 on all other occasions). Adverse events of dermatitis, including alopecia and dermal thickening, were observed in both groups, although only in the lotilaner group on Day 0. The only incident of vomiting and diarrhoea was observed in a control group dog.

### Study 2

At 6 h post-treatment, all but two of the lotilaner group dogs were free of live fleas, and of these two dogs, one had only a single moribund flea. On Day 7, at 6 h after new infestations had been applied, both these dogs were free of fleas, and the only flea-positive dog on this occasion had a single moribund flea. Overall, at 6 h post-treatment on Day 0, and at 6 h post-infestation on all study days, as compared to the control group, in the lotilaner group there were statistically significant reductions in mean flea counts (*t*_(7)_ ≥ 9.7, *P* < 0.0001), ranging from 98.9 to 100% (Table 2). A mild dermatitis was observed in two control dogs.

### Study 3

At 8 h post-treatment, as compared to the untreated controls, geometric mean flea count reductions in the lotilaner group were 99.9% and at least 99.5% at each subsequent infestation through Day 35 (Table 3). On Day 21, only a single flea, moribund, was found on a treated dog. On Day 28, a single moribund flea was found on two lotilaner-treated dogs, and another dog had one flea showing normal movement and one moribund flea. On Day 35, fleas showing normal movement were retrieved from two dogs, one with one flea, the other with two fleas. At 12 h post-treatment, and at all subsequent weekly infestations through Day 35, mean reductions in lotilaner-group counts were 100%, as all fleas had been killed (Table 4). In the eight-hour and 12-h groups, mean flea counts in the lotilaner group were significantly lower than those in the control group (*t*_(5)_ ≥ 12.3, *P* < 0.0001) at every assessment. Mild signs of diarrhoea were reported in both groups at various points in the study, and there was an incident of moderate diarrhoea and vomiting in a control dog. There were isolated incidents of dermatitis, including erythema and pruritus, observed predominantly in control group dogs, in some of which the condition was ongoing at the end of the study.

### Study 4

At 12 and 24 h post-treatment, and at all subsequent post-treatment assessments from weekly infestations through Day 35, mean reductions in lotilaner-group counts were 99.8 to 100% (Tables 5 and 6). In both time groups (i.e. those in which counts were completed at 12 and 24 h post-treatment, and post each weekly new infestations), mean flea counts in the lotilaner group were significantly lower than those in the control group (*t*_(7)_ ≥ 32.1, *P* < 0.0001). There were no adverse events attributed to lotilaner treatment.

## Discussion

Flea feeding can begin within minutes of the flea infesting the canine host, and flea egg production within 24 to 36 h of the first blood meal [5, 15]. The aim of a flea control program should be to minimize a dog’s exposure to the flea’s salivary antigens; to reduce the time of flea feeding and risk of transfer of pathogens from flea to host; and to prevent egg laying and thus contribute to a depletion of the flea stages in a dog’s environment. The speed of flea knockdown from the time of product administration that is reliably maintained through the next scheduled dose of insecticide is, therefore, an important consideration in choosing an appropriate flea control product.

The authors are aware of the variation between Studies 1–3 and Study 4 as to whether or not moribund fleas were counted separately, and whether or not they were included as live or dead in the efficacy assessments. This variation is because during the design of the studies there were changing approaches on the part of regulatory authorities concerning the classification of moribund fleas as live or dead. Following completion of these studies, health authorities in both the European Union and the USA have adopted a common position in regarding counting moribund fleas as “live”, which is how results have been presented for studies in which moribund fleas were counted separately.

The results of these studies met the expectations that were derived from pharmacokinetic work with lotilaner in fed dogs from two important aspects including the speed of onset of activity and sustained SOK. For SOK, rapid absorption of lotilaner produced peak blood levels within approximately 2 h after administration [11]. In the studies reported here, this rapid absorption translated into SOK efficacy of 89.9% by 4 h post-treatment.

The sustained speed of kill can be linked to lotilaner’s half-life, found to be approximately 30 days, ensuring that flea-lethal blood levels are maintained throughout and beyond the recommended monthly between-treatment interval [11]. In the duration-of-activity studies reported here, through weekly challenges up to 35 days after treatment, geometric mean flea count reductions compared to untreated controls were 97.8% within 4 h, while at 12 h following these challenges, efficacy remained at 100% at every post-treatment assessment through Day 35. Such persistence of rapid flea kill provides reassurance to veterinarians and pet owners that newly acquired fleas will continue to be rapidly killed beyond the end of the dosing period. These properties of lotilaner generate confidence that monthly treatments will disrupt the flea life-cycle, and that there will be no “end-of-dose” resurgence in flea burdens with a potential consequence of flares due to flea-bite hypersensitivity.

Across all studies, the absence of treatment-related adverse events indicates that lotilaner is well tolerated in dogs. The adverse events that were observed in study dogs occurred at a similar rate in treated and control groups, with the exception of skin and appendage disorders which were observed more frequently in the controls. This is presumably because of the effects of flea infestations which would have become established on dogs in the untreated groups, but because of treatment, failed to establish on lotilaner-treated dogs.

## Conclusions

The four studies described in this paper demonstrate that lotilaner provides a rapid onset of action against existing flea infestations on dogs, and sustains a rapid SOK for 35 days after treatment. Compared to control dogs, oral administration of lotilaner at a minimum dose of 20 mg/kg resulted in a significant reduction in mean flea counts of 89.9% at 4 h post-treatment, 99.2% at 6 h and 99.9% and 100% at eight and 12 h post-treatment, respectively. At each weekly assessment, lotilaner efficacy against newly infesting fleas at 4 h remained at > 97%, at 8 h remained at > 99%, and at 12 h remained at 100% through Day 35. Since fleas require 24 to 36 h after feeding to begin laying eggs, and lotilaner kills 100% of fleas within 12 h, this action of lotilaner can prevent new infestations and disrupt the flea life cycle in a contaminated environment, and its use offers a reliable means of rapidly eliminating the health risks that accompany flea infestations on dogs. The absence of treatment-related adverse effects in any of these studies further demonstrates lotilaner’s safety in dogs.

## Additional files

Additional file 1: Spanish translation of the article. (PDF 137 kb)

Additional file 2: French translation of the Abstract. (PDF 36 kb)

## Abbreviations

ANOVA: Analysis of variance
GABA: γ-aminobutyric acid
Mc: Mean number of live fleas in the untreated control group at each time point on each day
Mt.: Mean number of live fleas in the corresponding treated group at each time point on each day
SD: Standard deviation
SOK: Speed of kill
WAAVP: World Association for the Advancement of Veterinary Parasitology

## References

[1] McCoy C, Broce AB, Dryden MW (2008). Flea blood feeding patterns in cats treated with oral nitenpyram and the topical insecticides imidacloprid, fipronil and selamectin. Vet Parasitol.

[2] Varloud M, Fourie JJ (2015). Onset of efficacy and residual speed of kill over one month of a topical dinotefuran-permethrin-pyriproxyfen combination (Vectra® 3D) against the adult cat flea (*Ctenocephalides felis felis*) on dogs. Vet Parasitol.

[3] Dryden MW, Ryan WG, Bell M, Rumschlag AJ, Young LM, Snyder DE (2013). Assessment of owner-administered monthly treatments with oral spinosad or topical spot-on fipronil/(S)-methoprene in controlling fleas and associated pruritus in dogs. Vet Parasitol.

[4] Snyder DE, Rumschlag AJ, Young LM, Ryan WG (2015). Speed of flea knockdown of spinosad compared to afoxolaner, and of spinosad through 28 days post-treatment in controlled laboratory studies. Parasit Vectors.

[5] Blagburn BL, Dryden MW (2009). Biology, treatment, and control of flea and tick infestations. Vet Clin Small Anim.

[6] Bravecto® Freedom of Information Summary. https://www.fda.gov/downloads/animalveterinary/products/approvedanimaldrugproducts/foiadrugsummaries/ucm399075.pdf. Accessed 20 Jan 2017.

[7] McTier TL, Chubb N, Curtis MP, Hedges L, Inskeep GA, Knauer CS (2016). Discovery of sarolaner: A novel, orally administered, broad-spectrum, isoxazoline ectoparasiticide for dogs. Vet Parasitol.

[8] Shoop WL, Hartline EJ, Gould BR, Waddell ME, McDowell RG, Kinney JB (2014). Discovery and mode of action of afoxolaner, a new isoxazoline parasiticide for dogs. Vet Parasitol.

[9] Ozoe Y, Asahi M, Ozoe F, Nakahira K, Mita T (2010). The antiparasitic isoxazoline A1443 is a potent blocker of insect ligand-gated chloride channels. Biochem Biophys Res Commun.

[10] Kuntz EA, Kammanadiminti S. Safety evaluation of lotilaner in dogs after oral administration as flavoured chewable tablets (Credelio™). Parasit Vectors. 2017. (In press). 10.1186/s13071-017-2468-y PMC5664904 29089043

[11] Toutain CE, Seewald W, Jung M. The intravenous and oral pharmacokinetics of lotilaner in dogs. Parasit Vectors. 2017. (In press). 10.1186/s13071-017-2475-z PMC5664907 29089051

[12] Six RH, Liebenberg J, Honsberger NA, Mahabir SP (2016). Comparative speed of kill of sarolaner (Simparica) and afoxolaner (NexGard) against induced infestations of *Ctenocephalides felis* on dogs. Parasit Vectors.

[13] Beugnet F, Doyle V, Murray M, Chalvet-Monfray K (2011). Comparative efficacy on dogs of a single topical treatment with the pioneer fipronil/(S)-methoprene and an oral treatment with spinosad against *Ctenocephalides felis*. Parasite.

[14] Beugnet F, Liebenberg J, Halos L (2015). Comparative speed of efficacy against *Ctenocephalides felis* of two oral treatments for dogs containing either afoxolaner or fluralaner. Vet Parasitol.

[15] Rust MK, Dryden MW (1997). The biology, ecology and management of the cat flea. Annu Annu Rev Entomol.

