# Supplementary material for: Assessment of the speed of flea kill of lotilaner (Credelio™) throughout the month following oral administration to dogs
Source: Parasit Vectors. 2017 Nov 1;10:529. doi: 10.1186/s13071-017-2466-0 (PMC5664906; doi:10.1186/s13071-017-2466-0)
Supplement: Supplementary file 1 — Spanish translation of the article. (PDF 137 kb) [file 13071_2017_2466_MOESM1_ESM.pdf]

Evaluación de la velocidad para matar pulgas de lotilaner (Credelio^{TM}) a lo largo de un mes después de la administración oral en perros

Daniela Cavalleri[1], Martin Murphy[1], Wolfgang Seewald[1], Jason Drake[2]* y Steve Nanchen[1]

[1]Elanco Animal Health, Schwarzwaldallee 215, CH-4058 Basel WRO-1032.2.58, Suiza
[2]Elanco Animal Health, 2500 Innovation Way, Greenfield, IN 46140, USA

*Correspondencia, drake_jon_j@elanco.com

Correos electrónicos:
Daniela Cavalleri: cavalleri_daniela_a@elanco.com; Martin Murphy: murphy_martin_gerard@elanco.com; Wolfgang Seewald: seewald_wolfgang@elanco.com; Jason Drake: drake_jon_j@elanco.com; Steve Nanchen: nanchen_steve@elanco.com

Resumen

Antecedentes: Lotilaner (Credelio^{TM}, Elanco), una isoxazolina nueva, es un insecticida y acaricida sistémico que se absorbe rápidamente después de la administración oral a perros y tiene una vida media de 30 días. Como parte de un programa de desarrollo, los estudios se llevaron a cabo para investigar la eficacia inicial y sostenida de lotilaner y la velocidad para matar a las pulgas.

Métodos: Se condujeron cuatro estudios para evaluar el inicio de la velocidad de lotilaner para acabar con las pulgas al momento del tratamiento, y determinar la velocidad sostenida de matar (SOK) hasta por 35 días post-tratamiento y después de re-infestaciones semanales. En cada estudio, los perros se ubicaron aleatoriamente en un grupo tratados con lotilaner o en uno no tratado basándose en los conteos de pulgas pre-administración, antes del tratamiento se infestaron con *Ctenocephalides felis* adultas. Los perros que aleatoriamente se asignaron al grupo de lotilaner recibieron un solo tratamiento en el Día 0, a la dosis mínima recomendada a razón de 20 mg/kg, 30 (± 5) minutos después de haber sido alimentados. Se calculó la eficacia usando la media geométrica y aritmética de las cuentas de pulgas en los grupos tratados versus los no tratados.

Resultados: En el Día 0, la eficacia de lotilaner fue de 89.9% a las cuatro horas, 99.2% a las seis horas, 99.9% a las ocho horas, y 100% a las 12 horas post-tratamiento. En cada evaluación seminal,

la eficacia de lotilaner a las cuatro horas permaneció en > 97%, a las ocho horas permaneció en > 99%, y a las 12 horas permaneció a 100% hasta el Día 35. A lo largo de todos los estudios, no hubo eventos adversos relacionados con el tratamiento.

Conclusión: El rápido poder de eliminar a las pulgas de lotilaner inmediatamente después del tratamiento y el SOK sostenido a lo largo de 35 días post-tratamiento ofrece una nueva solución para ayudar a eliminar los riesgos de salud que acompañan a las infestaciones de pulgas en los perros. La consistencia de la rápida SOK sostenida para pulgas demostrada en estos estudios genera la confianza del uso mensual de lotilaner en perros puede ser valioso al interrumpir el ciclo de vida de la pulga en un ambiente contaminado, y que las pulgas recientemente adquiridas van a morir rápidamente, de ahí que se reduce el disconfort de la mordedura de la pulga. El SOK sostenido de lotilaner también da la confianza de que no habrá el resurgimiento en las cargas de pulgas “al final-de-la-dosis” con la consecuencia potencial que lo acompaña de las erupciones por hipersensibilidad a la mordedura de pulga.

Palabras clave: Pulgas, *Ctenocephalides felis*, Lotilaner, Credelio™, Velocidad para matar, Perro, Oral

Antecedentes

Los métodos para el control mensual de la pulga canina han pasado por cambios durante los últimos diez años, acelerando la tendencia a alejarse de la aplicación de los ectoparasiticidas spot-on y cambiando a favor del rango de productos administrados oralmente. En los primeros trabajos se demostró que los productos administrados oralmente tienen el potencial de matar a las pulgas más rápidamente que aquellos que actúan tópicamente, quizá debido al tiempo requerido para que un producto tópico se disperse del sitio de aplicación a las partas distales del animal [1, 2]. El surgimiento de los productos que matan pulgas que son administrados oralmente también han atraído la atención a otras deficiencias potenciales de los productos tópicos. Estos incluyen la preocupación acerca de la exposición de plaguicidas en el hogar, el requerimiento para la aplicación cuidadosa por los propietarios directamente en la piel de la mascota, y la disminución en las concentraciones de la piel durante el siguiente mes de tratamiento, que puede variar de animal en animal, y ser impredecible bajo las diferentes condiciones climáticas o exposición al agua [3].

Se ha demostrado que el primer producto adulticida contra pulgas de administración mensual oral para perros, el spinosad, puede proveer un rápido inicio de la eliminación de las pulgas

mientras provee una actividad de matar-pulgas de un mes [4]. El matar rápido a las pulgas se vio como algo deseable, no sólo para romper el ciclo de vida de la pulga, sino para prevenir la producción de huevos y remover la fuente de irritación causada por la mordedura, una causa de dermatitis alérgica, y matar rápidamente a las pulgas que ahora se sabe que transmiten varios agentes infecciosos tales como *Dipylidium caninum, Acanthocheilonema reconditum*, *Bartonella* spp. y *Rickettsia* spp. [5].

En una innovación más reciente en el control de ectoparásitos caninos ha surgido una nueva familia de compuestos, las isoxazolinas, que cuando se administran oralmente se ha demostrado que proveen una actividad sostenida contra las pulgas, así como contra algunas especies comunes de garrapatas [6-8]. Los primeros de estos compuestos que fueron aprobados están el afoxolaner y el fluralaner, en el 2014, seguidos, por el sarolaner, en el 2016. Se ha demostrado que las isoxazolinas tienen un modo de acción novedoso, uniéndose a los canales del ion cloro que son abiertos por el ácido γ-aminobutírico (GABA) y los receptores de glutamato, llevando a una parálisis progresiva e irreversible en insectos y ácaros [8, 9]. El sitio de unión de las isoxazolinas es diferente a la de otros insecticidas y acaricidas, y por lo tanto, la resistencia cruzada es poco probable que se desarrolle [8, 9].

La isoxazolina más reciente a aprobarse para uso en perros es el lotilaner. La molécula de lotilaner se seleccionó de una biblioteca de más de 500 compuestos debido a su potencial para proporcionar una eliminación rápida y sostenida de insectos y ácaros. Después de su selección y estudios preliminares, lotilaner se formula para desarrollar tabletas masticables sabor izadas (Credelio™, Elanco). Las investigaciones preclínicas incluyeron un estudio en el que se demostró que lotilaner es seguro cuando se administra en ocho ocasiones consecutivas mensualmente, en el punto más alto del rango de la dosis de etiqueta (20 a 43 mg/kg), y de tres- a cinco- veces la dosis para cachorros de ocho semanas de edad [10]. En un estudio farmacocinético cuando lotilaner se administró con alimento se vio que se absorbió rápidamente, con valores pico en plasma presentándose al cabo de aproximadamente dos horas después de la administración oral, y la vida media de aproximadamente 30 días [11].

Estas características estimularon las expectativas de que lotilaner proveería una eliminación rápida de las pulgas y garrapatas, y que la eliminación rápida podía ser sostenida por al menos un mes después de la administración, evitando cualquier preocupación de que su efectividad disminuya al final-de-la-dosis. Para evaluar la alineación de las expectativas de los atributos del producto con la realidad, se llevó a cabo una serie de cuatro estudios para investigar la eficacia de lotilaner contra pulgas (*Ctenocephalides felis*). Estos estudios fueron diseñados para proveer la información acerca

de la velocidad para matar (SOK) de lotilaner en el momento del tratamiento y hasta por 35 días después del tratamiento.

Mientras que el inicio de la acción de los productos de control de pulgas es importante para aliviar rápidamente la irritación causada por las infestaciones existentes, es también muy importante que la velocidad para matar (SOK) sea sostenida a lo largo de todo el período de dosificación indicado en la etiqueta. Se ha reportado el potencial de la reducción en la velocidad para matar a las pulgas al final-de-la-dosis en algunos productos, en donde las concentraciones del ingrediente activo disminuyen a niveles por debajo de aquellos necesarios para asegurar una efectividad insecticida rápida [12-14].

Métodos

Los estudios reportados en este artículo fueron llevados a cabo en cuatro laboratorios en tres países. Todo el personal que condujo las observaciones del estudio y los conteos o que llevaron a cabo las infestaciones fueron ciegos a los tratamientos asignados.

Animales

Los perros fueron Beagle sanos de raza pura, machos y hembras de una edad entre los siete meses y los nueve años, con rangos de peso de 6.8 a 19.6 kg. Los perros que se enrolaron se seleccionaron de un grupo más grande que se había aclimatizado a las instalaciones de cada estudio respectivo, y conforme fue necesario todos pasaron por un período de lavado suficiente para asegurar que no había ningún residuo de parasiticida presente que pudiera afectar los resultados del estudio. En todos los estudios, los perros se alojaron en corrales individuales, sin contacto directo posible entre perros individualmente. Se tomaron las precauciones estándar para asegurar que no hubo contaminación cruzada entre perros dentro o entre los grupos de tratamiento. A los perros se les proporcionó agua y alimento de acuerdo con el procedimiento estándar de cada instalación, y las observaciones de salud se hicieron al menos una vez al día a lo largo de los períodos pre-estudio y estudio. Al final del estudio, los perros fueron regresados a las perreras de estudio.

A lo largo de cada estudio, las observaciones se hicieron para determinar y registrar la presentación de cualquier evento adverso. Un evento adverso se define como cualquier observación de algún signo clínico en algún perro que fuera desfavorable y no intencionado, y que ocurriera

después del uso del producto, independientemente que el evento se considerara relacionado o no con el producto [15].

Diseño

Todos los estudios utilizaron un diseño aleatorio, ciego, con control negativo. Para los propósitos de la aleatorización, los perros se infestaron con hasta, aproximadamente, 100 pulgas entre seis días y dos semanas antes de comenzar cada estudio, las pulgas se sacaron con peine y se contaron 24 ó 48 horas post-infestación. Estos conteos se utilizaron para clasificar a los perros, siendo que aquellos que no retuvieran al menos 50% de la infestación eran inelegibles para ser incluidos en el estudio. Como no hay efecto de sexo sobre la absorción sistémica de lotilaner, esto no fue un factor para la clasificación y la aleatorización [10]. Se utilizó la aleatorización para colocar a los perros en bloques, y dentro de cada bloque los perros se ubicaron aleatoriamente en un grupo tratado o en un grupo control. En todos los estudios, la comparación se hizo contra un grupo control tratado sin efecto o no tratado, y todos los perros consumieron una ración diaria parcial al cabo de, aproximadamente, 30 minutos antes de administrar el tratamiento. Hubo ocho perros incluidos en cada grupo de estudio dentro de los cuatro estudios.

Tratamiento, infestaciones por pulga y peinado

Cada estudio incluyó al menos un grupo en donde lotilaner se administró oralmente a una dosis mínima de 20 mg/kg y al menos un grupo control correspondiente que recibió el producto antiparasitario no activo. Todos los perros habían consumido una ración parcial diaria al cabo de, aproximadamente, 30 minutos antes de la dosificación. El número de grupos en cada estudio se fijó de acuerdo al número de intervalos de tiempo en donde se llevaron a cabo las evaluaciones. Los grupos control contemporáneos se usaron en cada punto de tiempo evaluado.

- ***Estudio 1*****:** Un grupo tratado con lotilaner, y un grupo control. Los conteos de pulgas se llevaron a cabo cuatro horas post-dosificación contra una infestación existente y cuatro horas después de las nuevas infestaciones en los días 7, 14, 21, 28 y 35
- ***Estudio 2*****:** Un grupo tratado con lotilaner, y un grupo control. Los conteos de pulgas se llevaron a cabo a las seis horas post-dosificación contra la infestación existente y seis horas después de las nuevas infestaciones en los días 7, 14, 21, 28 y 35

- ***Estudio 3*****:** Dos grupos tratados con lotilaner, y dos grupos control. Los conteos de pulgas se llevaron a cabo a las ocho y 12 horas post-dosficación contra la infestación de pulgas existente y ocho y 12 horas después de nuevas infestaciones en los días 7, 14, 21, 28 y 35
- ***Estudio 4*****:** Dos grupos tratados con lotilaner, y dos grupos control. Los conteos de pulgas se llevaron a cabo a las 12 y 24 horas post-dosificación contra una infestación existente de pulgas y a las 12 y 24 horas después de nuevas infestaciones en los días 7, 14, 21, 28 y 35

Las infestaciones con las pulgas se llevaron a cabo de acuerdo al procedimiento estándar de cada laboratorio, típicamente involucrando una aplicación a partir de un vial que contenía 100 pulgas adultas, recientemente eclosionadas, no alimentadas colocadas en la base de la cola o en un flanco del perro. Las cepas de las pulgas eran originarias de Irlanda, Alemania-Dinamarca y de los Estados Unidos. Se completó el peinado para el conteo de pulgas en tiempos especificados por el protocolo usando peines para pulga con dientes finos para determinar la eficacia del producto. Las técnicas de recuperación de pulgas se aplicaron de acuerdo al procedimiento estándar de cada laboratorio, con cierta variación entre laboratorios. En dos estudios, se requirió que el peinado durara al menos cinco minutos, extendiéndose más allá del tiempo si continuaban encontrándose pulgas. En dos estudios, se requirió un mínimo de 10 minutos, y se extendieron dos minutos adicionales si se encontraban pulgas, a un máximo de 16 minutos.

En todos los estudios, las pulgas recuperadas se clasificaron como vivas o mueras; una pulga se clasificó como viva si ésta se podía mover activamente entre el pelo, y sí se colocaba sobre una superficie plana, y rápidamente se "enderezaba" y se movía fácilmente o brincaba. Del estudio 1 al 3, cualquier pulga que estuviera en decúbito lateral y que no se podía mover normalmente entre el pelo o enderezarse cuando se colocaba en una superficie plana, pero todavía tenía movimientos de patas o se retorcía era registrada como moribunda pero se contaba entre los conteos de pulgas vivas. Una pulga se clasificaba como muera si estaba completamente inmóvil. En el estudio 4 las pulgas moribundas no se contaban por separado y se incluyeron como muertas.

Evaluaciones de eficacia

El perro individual fue la unidad experimental. Usando la fórmula a continuación, se calculó la eficacia contra las pulgas por separado para cada conteo en un punto de tiempo. Todos los análisis se llevaron a cabo utilizando el procedimiento PROC MIXED (SAS 9.2, Cary NC). Se calcularon las medias geométricas usando los conteos transformados logarítmicamente (conteo + 1) con uno (1)

subsecuentemente restado del resultado, y un modelo ANOVA se usó para comparar a los grupos tratados de los no tratados.

$$\text{Eficacia (\%)} = 100 \times (Mc - Mt)/Mc$$

En dónde: Mc es el número medio de pulgas vivas en el grupo control no tratado en cada punto de tiempo en cada día, y Mt es el número medio de pulgas vivas en el grupo tratado correspondiente en cada punto de tiempo en cada día.

El tratamiento se consideró efectivo en cualquier evaluación si al menos seis animales en los grupos control respectivos tuvieron una tasa de recuperación de al menos un 50%, aproximadamente, si relativo al grupo control el porcentaje de reducción en los conteos medios de pulgas en el grupo tratado fue de al menos 90% (o 95%, dependiendo del requerimiento regulatorio), y sí la reducción fue estadísticamente significativa ($P < 0.05$).

Resultados

Las infestaciones en los grupos control en cada punto de tiempo y en cada estudio fueron adecuadas. La eficacia basada en las medias geométricas fue del 89.9% a las cuatro horas post-tratamiento, y a las cuatro horas después de las infestaciones subsecuente tuvo un rango de 97.8 a 100% hasta y a lo largo del Día 35. A las ocho horas post-tratamiento y después de las nuevas infestaciones, la eficacia de lotilaner fue de al menos 99.5% hasta y a lo largo del Día 35, y a las 12 horas fue del 100% en todas las evaluaciones hasta el Día 35 (Fig. 1; Tablas 1–6).

Estudio 1

Al cabo de cuatro horas después del tratamiento, la media geométrica del conteo de pulgas se había reducido en un 89.9% en los perros tratados con lotilaner; y después de todas las infestaciones subsecuentes, las reducciones en el conteo de pulgas a las cuatro horas tanto en las medias geométricas, como en las aritméticas estuvieron dentro del rango de eficacia del 97.1 al 100% (Tabla 1). Las pulgas moribundas estuvieron presentes en los perros del grupo de lotilaner en todos los días, excepto en el Día 14 en donde no se clasificaron pulgas como vivas o moribundas. Los números de pulgas moribundas en los perros tratados con lotilaner variaron entre los desafíos, con un total de 20 en el Día 0, y en un rango de 1 a 14 en los otros días. En comparación con el grupo

control, las reducciones en el conteo medio de pulgas para el grupo de lotilaner fue significativo en cada evaluación hasta el Día 35 ($t_{(5)} = 3.4$, $P = 0.0194$ en Día 0 y $t_{(5)} \geq 14.9$, $P < 0.0001$ en todas las otras ocasiones). Los eventos adversos de dermatitis, incluyendo alopecia y engrosamiento dérmico, se observaron en ambos grupos, aunque solo en el grupo de lotilaner en el Día 0. El único incidente de vómito y diarrea se observe en un perro del grupo control.

Estudio 2

A las seis horas post-tratamiento, todos, excepto dos perros del grupo de lotilaner estuvieron libres de pulgas, y uno de esos dos perros, tuvo una sola pulga moribunda. En el Día 7, a las seis horas después de que las nuevas infestaciones se aplicaran, ambos perros estaban libres de pulgas, y el único perro positivo a pulgas en esta ocasión tuvo una sola pulga moribunda. En general, a las seis horas post-tratamiento en el Día 0, y a las seis horas post-infestación en todos los días del estudio, en comparación con el grupo control, el grupo de lotilaner tuvo reducciones estadísticamente significativas en los conteos medios de pulgas ($t_{(7)} \geq 9.7$, $P < 0.0001$), con un rango de 98.9 a 100% (Tabla 2). Se observó una leve dermatitis en dos perros control.

Estudio 3

A las ocho horas post-tratamiento, en comparación con los controles no tratados, las reducciones en la media geométrica del conteo de pulgas en el grupo de lotilaner fueron del 99.9% y al menos 99.5% en cada infestación subsecuente hasta el Día 35 (Tabla 3). En el Día 21, solamente una sola pulga, moribunda se encontró en un perro tratado. En el Día 28, una sola pulga moribunda se encontró en dos perros tratados con lotilaner, y otro perro tuvo una pulga que mostró movimiento normal y una pulga moribunda. En el Día 35, las pulgas que mostraban un movimiento normal se recuperaron de dos perros, uno con una pulga y el otro con dos pulgas. A las 12 horas post-tratamiento, y en todas las infestaciones subsecuentes semanales hasta el Día 35, las reducciones en la media del conteo de pulgas en el grupo de lotilaner fueron del 100%, ya que todas las pulgas estaban muertas (Tabla 4). En los grupos de ocho horas y 12 horas, los conteos medios de pulgas en el grupo de lotilaner fueron significativamente más bajos que aquellos en el grupo control ($t_{(5)} \geq 12.3$, $P < 0.0001$) en cada evaluación. Se reportaron signos leves de diarrea en ambos grupos en varios puntos del estudio, y hubo un incidente de diarrea moderada y vómito en un perro control. Hubo incidentes aislados de dermatitis, incluyendo eritema y prurito, observados predominantemente en el grupo control de perros, en algunos de los cuáles la condición continuó hasta el final del estudio.

Estudio 4

A las 12 y 24 horas post-tratamiento, y en todas las evaluaciones subsecuentes post-tratamiento de las infestaciones semanales hasta el Día 35, las reducciones medias en los conteos del grupo de lotilaner fueron del 99.8 al 100% (Tablas 5, 6). En ambos grupos de tiempo (i.e. aquellos en los que los conteos se completaron a las 12 y 24 horas post-tratamiento, y después de cada infestación nueva semanal), los conteos medios de pulgas en el grupo de lotilaner fueron significativamente menores que aquellos en el grupo control ($t_{(7)} \geq 32.1$, $P < 0.0001$). No hubo eventos adversos atribuidos al tratamiento con lotilaner.

Discusión

La alimentación de la pulga puede comenzar en minutos después de que la pulga ha infestado al hospedador canino, y la producción de huevos al cabo de 24 a 36 horas de la primera comida de sangre [5, 15]. El objetivo del programa de control de pulgas deberá de minimizar la exposición del perro a los antígenos salivales de la pulga; deberá reducir el tiempo de alimentación de la pulga y el riesgo de transferir patógenos de la pulga al hospedador; y deberá de prevenir la postura de huevos y por ende, contribuir a la depleción de las etapas de la pulga en el ambiente del perro. La velocidad de eliminar pulgas desde el momento en que se administra un producto que sea confiablemente mantenido hasta la siguiente dosis programada del insecticida es, por lo tanto, una consideración muy importante para escoger el producto para el control de pulgas apropiado.

Los autores están conscientes de la variación entre los Estudios 1-3 y el Estudio 4, en lo que respecta a sí las pulgas moribundas se contaron por separado, y sí se incluyeron como vivas o muertas en las evaluaciones de eficacia. Esta variación se debe a que durante el diseño de los estudios hubo enfoques cambiantes por parte de las autoridades regulatorias en lo que se refiere a la clasificación de pulgas moribundas como vivas o muertas. Después de completar estos estudios, las autoridades de salud tanto en la Unión Europea y en E.U.A han adoptado una posición común referente al conteo de pulgas moribundas como “vivas”, que es como se han presentado los resultados de estos estudios, en los que las pulgas moribundas se contaron por separado.

Los resultados de estos estudios cubren las expectativas derivadas del trabajo de farmacocinética con lotilaner en perros alimentados en dos aspectos importantes incluyendo la velocidad del inicio de la actividad y la SOK sostenida. Para la SOK, la rápida absorción de

lotilaner produjo niveles pico en sangre al cabo aproximadamente de dos horas después de la administración [11]. En los estudios reportados aquí, esta rápida absorción se tradujo en la eficacia de SOK del 89.9% a las cuatro horas post-tratamiento.

La velocidad para matar sostenida puede relacionarse a la vida media de lotilaner que se ha visto que es de aproximadamente 30 días, asegurando que los niveles letales en sangre para la pulga se mantengan a lo largo y más allá del intervalo entre tratamientos recomendado mensualmente [11]. En los estudios de duración-de-actividad reportados aquí, a través de desafíos semanales hasta 35 días después del tratamiento, las reducciones en la media geométrica del conteo de pulgas comparada con los controles no tratados fueron de 97.8% al cabo de cuatro horas, mientras que a 12 horas después de estos desafíos, la eficacia permaneció a 100% en cada evaluación post-tratamiento hasta el Día 35. Dicha persistencia de matar rápido a las pulgas le proporciona a los veterinarios y a los propietarios de las mascotas el reaseguramiento de que las pulgas recién adquiridas continuarán siendo matadas rápidamente, más allá del final del período de dosificación. Estas propiedades de lotilaner generan confianza de que los tratamientos mensuales van a interrumpir el ciclo de vida de la pulga, y que no hay resurgimiento “al-final-de-la-dosis” en las cargas de pulgas con la consecuencia potencial de erupciones debido a la hipersensibilidad por mordedura de pulga.

En todos los estudios, la ausencia de eventos adversos en relación con el tratamiento indica que lotilaner es bien tolerado en perros. Los eventos adversos que se observaron en los perros del estudio se presentaron en un nivel similar en los grupos tratados y los grupos control, con excepción de desórdenes de piel y apéndices que se observaron más frecuentemente en los controles. Esto es posiblemente debido a los efectos de las infestaciones por pulga que se han establecido en los perros de los grupos no tratados, pero debido al tratamiento, no se establecieron en los perros tratados con lotilaner.

Conclusiones

Los cuatro estudios descritos en este artículo demostraron que lotilaner provee un rápido inicio de la acción contra las infestaciones de pulgas existentes en los perros, y mantiene una SOK rápida durante 35 días después del tratamiento. En comparación con los perros control, la administración oral de lotilaner a una dosis mínima de 20 mg/kg resultó en una reducción significativa en la media de los conteos de pulgas de un 89.9% a las cuatro horas post-tratamiento, 99.2% y 100% a las ocho y 12 horas post-tratamiento, respectivamente. En cada evaluación semanal, la eficacia de lotilaner contra las infestaciones recientes de pulgas a las 4 horas permaneció en > 97%, a las ocho horas en >

99%, y a las 12 horas permaneció al 100% hasta el Día 35. Puesto que las pulgas requieren de 24 a 36 horas después de la alimentación para comenzar a poner huevos, y lotilaner mata el 100% de las pulgas al cabo de 12 horas, esta acción de lotilaner puede prevenir nuevas infestaciones e interrumpir el ciclo de vida de la pulga en un ambiente contaminado, y su uso ofrece un medio más confiable para eliminar los riesgos de salud que acompañan a las infestaciones por pulgas en los perros. La ausencia de efectos adversos en relación con el tratamiento en todos estos estudios demostró la seguridad de lotilaner en perros.

Abreviaturas

ANOVA: análisis de varianza; GABA:ácido γ-aminobutírico; Mc: media del número de pulgas vivas en el grupo control no tratado en cada punto de tiempo en cada día; Mt: media del número de pulgas vivas en el grupo de tratado correspondiente en cada punto de tiempo en cada día; SD: Desviación estándar; SOK: Velocidad para matar; WAAVP: Asociación Mundial para los Avances de Parasitología Veterinaria

Reconocimientos

Los autores quieren agradecer al Dr. Bill Ryan de Ryan Mitchell Associates, LLC por su apoyo con este manuscrito.

Declaración de ética y consentimiento para participar

Todos los estudios se completaron de acuerdo con las regulaciones locales, regionales y nacionales con los principios de Buenas Prácticas Clínicas, y con las guías de la Asociación Mundial para los Avances de Parasitología Veterinaria (WAAVP) para evaluar la eficacia de los parasiticidas para el tratamiento, prevención y control de infestaciones por pulgas y garrapatas en perros y gatos. El estudio fue aprobado por el Grupo de Bienestar Animal de Novartis Animal Health Global. .

Consentimiento para publicación

No aplica.

Disponibilidad de datos y material

Debido a la confidencialidad comercial de la investigación, los datos no incluidos en el manuscrito solo pueden estar disponibles para investigadores *bona fide* sujeto un acuerdo de no-divulgación.

Intereses competentes

DC, MM, WS, JD y SN son empleados de Elanco Animal Health.

Fondos

Todos los estudios fueron patrocinados por Elanco.

Contribuciones de los autores

Todos los autores participaron en uno o más de los diseños, completaron y reportaron los estudios y se involucraron en la versión preliminar del manuscrito. Todos los autores leyeron y aprobaron el manuscrito final.

Detalles del autor

[1]Elanco Animal Health, Schwarzwaldallee 215, CH-4058 Basel WRO-1032.2.58., Suiza

[2]Elanco Animal Health, 2500 Innovation Way, Greenfield, IN 46140, E.U.A.

Referencias

1. McCoy C, Broce AB, Dryden MW. Flea blood feeding patterns in cats treated with oral nitenpyram and the topical insecticides imidacloprid, fipronil and selamectin. Vet Parasitol. 2008;156:293-301.
2. Varloud M, Fourie JJ. Onset of efficacy and residual speed of kill over one month of a topical dinotefuran-permethrin-pyriproxyfen combination (Vectra® 3D) against the adult cat flea (*Ctenocephalides felis felis*) on dogs. Vet Parasitol. 2015;211:89-92.
3. Dryden MW, Ryan WG, Bell M, Rumschlag AJ, Young LM, Snyder DE. Assessment of owner-administered monthly treatments with oral spinosad or topical spot-on fipronil/(S)-methoprene in controlling fleas and associated pruritus in dogs. Vet Parasitol. 2013;191:340-6.
4. Snyder DE, Rumschlag AJ, Young LM, Ryan WG. Speed of flea knockdown of spinosad compared to afoxolaner, and of spinosad through 28 days post-treatment in controlled laboratory studies. Parasit Vectors. 2015;8:578.
5. Blagburn BL, Dryden MW. Biology, treatment, and control of flea and tick infestations. Vet Clin Small Anim. 2009;39:1173–1200.

6. Bravecto® Freedom of Information Summary. http://www.fda.gov/downloads/AnimalVeterinary/Products/ApprovedAnimalDrugProducts/FOIADrugSummaries/UCM488816.pdf. Accessed January 20, 2017.
7. McTier TL, Chubb N, Curtis MP, Hedges L, Inskeep GA, Knauer CS, et al. Discovery of sarolaner: A novel, orally administered, broad-spectrum, isoxazoline ectoparasiticide for dogs. Vet Parasitol. 2016;222:3-11.
8. Shoop WL, Hartline EJ, Gould BR, Waddell ME, McDowell RG, Kinney JB, et al. Discovery and mode of action of afoxolaner, a new isoxazoline parasiticide for dogs. Vet Parasitol. 2014;201:179-89.
9. Ozoe Y, Asahi M, Ozoe F, Nakahira K, Mita T. The antiparasitic isoxazoline A1443 is a potent blocker of insect ligand-gated chloride channels. Biochem Biophys Res Commun. 2010;391:744-9.
10. Kuntz EA, Kammanadiminti S. Safety evaluation of lotilaner in dogs after oral administration as flavoured chewable tablets (Credelio™). Parasit Vectors (In press).
11. Toutain CE, Seewald W, Jung M. The intravenous and oral pharmacokinetics of lotilaner and the effect of food in dogs. Parasit Vectors (In press).
12. Six RH, Liebenberg J, Honsberger NA, Mahabir SP. Comparative speed of kill of sarolaner (Simparica) and afoxolaner (NexGard) against induced infestations of *Ctenocephalides felis* on dogs. Parasit Vectors. 2016;9:90.
13. Beugnet F, Doyle V, Murray M, Chalvet-Monfray K. Comparative efficacy on dogs of a single topical treatment with the pioneer fipronil/(S)-methoprene and an oral treatment with spinosad against *Ctenocephalides felis*. Parasite. 2011;18:325-31.
14. Beugnet F, Liebenberg J, Halos L. Comparative speed of efficacy against *Ctenocephalides felis* of two oral treatments for dogs containing either afoxolaner or fluralaner. Vet Parasitol. 2015;207:297-301.
15. Rust MK, Dryden MW. The biology, ecology and management of the cat flea. Annu Annu Rev Entomol. 1997:42:451-73.

Leyenda para la figura

Fig. 1 Porcentaje de reducciones en la media geométrica en los conteos de pulgas para lotilaner en comparación con los controles no tratados a las 4, 6, 8 y 12 horas post-tratamiento y en los mismos intervalos después de las infestaciones semanales hasta los 35 días después del tratamiento

Tabla 1 Resultados de las infestaciones de pulga a las cuatro horas post-tratamiento y cuatro horas después de las infestaciones semanales en el Estudio 1

		**Día de infestación y conteo de pulgas**					
		**0**	**7**	**14**	**21**	**28**	**35**
Control	Media aritmética ± SD	60.9 ± 12.5	76.0 ± 9.8	78.6 ± 13.1	71.0 ± 6.4	67.3 ± 5.6	73.1 ± 12.3
	Media geométrica	59.9	75.4	77.7	70.8	67.1	72.3
Lotilaner	Media aritmética ± SD	26.4 ± 33.8	0.1 ± 0.4	0.0 ± 0.0	1.0 ± 1.3	0.8 ± 1.2	2.1 ± 1.7
	Eficacia (%)	56.7	99.8	100	98.6	98.9	97.1
	Media geométrica	6.1	0.1	0.0	0.7	0.5	1.6
	Eficacia (%)	89.9	99.9	100	99.0	99.3	97.8
Comparación		$t_{(5)} = 3.4$, $P = 0.0194$	$t_{(5)} = 55.0$, $P < 0.0001$	$t_{(5)} = 91.5$, $P < 0.0001$	$t_{(5)} = 21.8$, $P < 0.0001$	$t_{(5)} = 18.5$, $P < 0.0001$	$t_{(5)} = 14.9$, $P < 0.0001$

Abreviaturas: SD, desviación estándar

Tabla 2 Resultados de las infestaciones de pulgas a las seis horas después de las infestaciones semanales en el Estudio 2

		**Día de infestación y conteo de pulgas**					
		**0**	**7**	**14**	**21**	**28**	**35**
Control	Media aritmética ± SD	76.6 ± 20.1	78.6 ± 8.7	88.9 ± 12.2	95.4 ± 5.6	94.5 ± 7.6	93.8 ± 7.9
	Media geométrica	74.3	78.2	88.1	95.2	94.2	93.5
Lotilaner	Media aritmética ± SD	2.6 ± 7.0	0.1 ± 0.4	0.0 ± 0.0	3.6 ± 10.3	1.6 ± 3.1	1.8 ± 2.4
	Eficacia (%)	96.6	99.8	100	96.2	98.3	98.1
	Media geométrica	0.6	0.1	0.0	0.5	0.8	1.0
	Eficacia (%)	99.2	99.9	100	99.4	99.1	98.9
Comparación		$t_{(7)} = 11.4$, $P < 0.0001$	$t_{(7)} = 45.0$, $P < 0.0001$	$t_{(7)} = 88.4$, $P < 0.0001$	$t_{(7)} = 9.7$, $P < 0.0001$	$t_{(7)} = 14.3$, $P < 0.0001$	$t_{(7)} = 13.6$, $P < 0.0001$

Abreviaturas: SD, desviación estándar

Tabla 3 Resultados de las infestaciones de pulgas a las ocho horas post-tratamiento después de las infestaciones semanales en el Estudio 3

		**Día de infestación y conteo de pulgas**					
		**0**	**7**	**14**	**21**	**28**	**35**
Control	Media aritmética ± SD	75.1 ± 6.7	82.3 ± 7.8	80.8 ± 11.4	81.4 ± 13.8	78.9 ± 13.4	76.3 ± 7.4
	Media geométrica	74.9	81.9	80.0	80.4	77.9	75.9
Lotilaner	Media aritmética ± SD	0.1 ± 0.4	0.0 ± 0.0	0.0 ± 0.0	0.1 ± 0.4	0.5 ± 0.8	0.4 ± 0.7
	Eficacia (%)	99.8	100	100	99.8	99.4	99.5
	Media geométrica	0.1	0.0	0.0	0.1	0.4	0.3
	Eficacia (%)	99.9	100	100	99.9	99.5	99.7
Comparación		$t_{(5)} = 46.0$, $P < 0.0001$	$t_{(5)} = 128.2$, $P < 0.0001$	$t_{(5)} = 40.4$, $P < 0.0001$	$t_{(5)} = 42.1$, $P < 0.0001$	$t_{(5)} = 12.3$, $P < 0.0001$	$t_{(5)} = 27.6$, $P < 0.0001$

Abreviaturas: SD, desviación estándar

Tabla 4 Resultados de las infestaciones de pulgas a las 12 horas post-tratamiento y a las 12 horas después de las infestaciones semanales en el Estudio 3

		**Día de infestación y conteo de pulgas**					
		**0**	**7**	**14**	**21**	**28**	**35**
Control	Media aritmética ± SD	75.0 ± 7.0	71.9 ± 9.2	76.9 ± 12.8	78.0 ± 9.4	75.1 ± 6.2	82.0 ± 10.3
	Media geométrica	74.7	71.4	75.9	77.6	74.9	81.4
Lotilaner	Media aritmética ± SD	0.0 ± 0.0	0.0 ± 0.0	0.0 ± 0.0	0.0 ± 0.0	0.0 ± 0.0	0.0 ± 0.0
	Eficacia (%)	100	100	100	100	100	100
	Media geométrica	0.0	0.0	0.0	0.0	0.0	0.0
	Eficacia (%)	100	100	100	100	100	100
Comparación		$t_{(5)} = 78.5$, $P < 0.0001$	$t_{(5)} = 92.6$, $P < 0.0001$	$t_{(5)} = 42.2$, $P < 0.0001$	$t_{(5)} = 112.6$, $P < 0.0001$	$t_{(5)} = 146.6$, $P < 0.0001$	$t_{(5)} = 99.0$, $P < 0.0001$

Abreviaturas: SD, desviación estándar

Tabla 5 Resultados de las infestaciones de pulgas a las 12 horas post-tratamiento y a las 12 horas después de las infestaciones semanales en el Estudio 4

		**Día de infestación y conteo de pulgas**					
		**0**	**7**	**14**	**21**	**28**	**35**
Control	Media aritmética ± SD	89.5 ± 36.9	69.8 ± 18.0	75.1 ± 22.6	75.8 ± 22.6	76.5 ± 19.4	59.8 ± 10.1
	Media geométrica	83.6	67.9	72.6	73.1	74.1	59.1
Lotilaner	Media aritmética ± SD	0.0 ± 0.0	0.0 ± 0.0	0.0 ± 0.0	0.0 ± 0.0	0.0 ± 0.0	0.0 ± 0.0
	Eficacia (%)	100	100	100	100	100	100
	Media geométrica	0.0	0.0	0.0	0.0	0.0	0.0
	Eficacia (%)	100	100	100	100	100	100
Comparación			$t_{(7)} = 49.7$, $P < 0.0001$	$t_{(7)} = 44.7$, $P < 0.0001$	$t_{(7)} = 43.5$, $P < 0.0001$	$t_{(7)} = 43.9$, $P < 0.0001$	$t_{(7)} = 75.0$, $P < 0.0001$

Abreviaturas: SD, desviación estándar

Tabla 6 Resultados de las infestaciones de pulgas a las 24 horas post-tratamietno y 24 horas después de las infestaciones semanales en el Estudio 4

		**Día de infestación (conteos de pulgas completados al día siguiente)**					
		**0**	**7**	**14**	**21**	**28**	**35**
Control	Media Aritmética ± SD	87.6 ± 7.4	72.1 ± 15.6	76.1 ± 22.4	65.6 ± 14.9	66.9 ± 18.3	59.5 ± 10.3
	Media Geométrica	87.4	70.7	73.6	64.3	65.2	58.6
Lotilaner	Media Aritmética ± SD	0.0 ± 0.0	0.0 ± 0.0	0.1 ± 0.4	0.1 ± 0.3	0.0 ± 0.0	0.0 ± 0.0
	Eficacia (%)	100	100	99.8	99.8	100	100
	Media Geométrica	0.0	0.0	0.1	0.1	0.0	0.0
	Eficacia (%)	100	100	99.8	99.8	100	100
Comparación			$t_{(7)} = 55.8$, $P < 0.0001$	$t_{(7)} = 38.7$, $P < 0.0001$	$t_{(7)} = 35.7$, $P < 0.0001$	$t_{(7)} = 52.4$, $P < 0.0001$	$t_{(7)} = 61.3$, $P < 0.0001$

Abreviaturas: SD, desviación estándar
