# Supplementary material for: Assessment of the speed of flea kill of lotilaner (Credelio™) throughout the month following oral administration to dogs
Source: Parasit Vectors. 2017 Nov 1;10:529. doi: 10.1186/s13071-017-2466-0 (PMC5664906; doi:10.1186/s13071-017-2466-0)
Supplement: Supplementary file 2 — French translation of the Abstract. (PDF 36 kb) [file 13071_2017_2466_MOESM2_ESM.pdf]

Évaluation de la rapidité d'action du lotilaner (Credelio™) contre les puces au cours du mois suivant son administration par voie orale chez le chien

Daniela Cavalleri[1], Martin Murphy[1], Wolfgang Seewald[1], Jason Drake[2]* et Steve Nanchen[1]

[1]Elanco Santé animale, Schwarzwaldallee 215, CH-4058 Bâle, WRO-1032.2.58, Suisse
[2]Elanco Santé animale, 2500 Innovation Way, Greenfield, IN 46140, États-Unis

*Correspondance : drake_jon_j@elanco.com

Adresse électronique :
Daniela Cavalleri : cavalleri_daniela_a@elanco.com ; Martin Murphy : murphy_martin_gerard@elanco.com ; Wolfgang Seewald : seewald_wolfgang@elanco.com ; Jason Drake : drake_jon_j@elanco.com ; Steve Nanchen : nanchen_steve@elanco.com

Résumé

Contexte : le lotilaner (Credelio™, Elanco) est un nouvel insecticide et acaricide systémique de la classe des isoxazolines qui est rapidement absorbé après administration orale et dont la demi-vie est de 30 jours. Des études ont été conduites dans le cadre d'un programme de développement afin d'explorer l'efficacité initiale et résiduelle du lotilaner et sa rapidité d'action (*« speed of kill »*) contre les puces.
Méthodes : quatre études ont été conduites afin de déterminer la rapidité d'apparition de l'effet « knockdown » sur les puces au moment du traitement et de déterminer la rémanence de cet effet létal jusqu’à 35 jours après le traitement. Des évaluations ont été réalisées à un ou deux moments spécifiques (4, 6, 8 et 12 heures) de chaque étude, après le traitement et suite à de nouvelles infestations hebdomadaires. Dans chaque étude, les chiens ont été randomisés dans un groupe lotilaner ou un groupe « non traité » selon un comptage des puces préalable à l'administration du traitement. Ils ont ensuite été infestés par des puces adultes de l'espèce *Ctenocephalides felis* avant de recevoir le traitement. Les chiens randomisés dans un groupe lotilaner ont reçu un seul traitement à J0 à la dose minimale recommandée de 20 mg/kg, 30 (± 5) minutes après avoir été nourris. L'efficacité était mesurée à l'aide des moyennes géométriques et arithmétiques du nombre de puces récoltées dans les groupes traités par rapport aux groupes non traités.

Résultats : à J0, l'efficacité du lotilaner a été de 89,9 % à quatre heures, 99,2 % à six heures, 99,9 % à huit heures et 100 % à 12 heures du traitement. Lors de chaque évaluation hebdomadaire, l'efficacité du lotilaner était systématiquement > 97 % à quatre heures, > 99 % à huit heures et de 100 % à 12 heures et ce jusqu'à J35. Aucun événement indésirable liés au traitement n'a été observé au cours d'aucune de ces études.

Conclusion : grâce à son effet « knockdown » rapide sur les puces immédiatement après administration et à la persistance de son activité pendant 35 jours après le traitement, le lotilaner apporte une nouvelle réponse dans la lutte contre les risques sanitaires associés aux infestations par les puces chez le chien. L'effet rapide et persistant contre les puces démontré dans chacune de ces études confirme l'intérêt d'une administration mensuelle de lotilaner chez le chien. Ce schéma d'administration doit non seulement permettre de rompre le cycle de vie des puces dans un environnement contaminé, mais aussi d'éliminer rapidement les puces nouvellement acquises et de réduire ainsi l'inconfort dû aux piqûres de puces. La persistance de cet effet létal rapide sur les puces doit permettre d'éviter la résurgence des infestations en fin d'intervalle posologique, et donc l'exacerbation de l'hypersensibilité aux piqûres de puces susceptible de l'accompagner.
